# Sexual Dimorphism in Lesion Size and Sensorimotor Responses Following Spinal Cord Injury

**DOI:** 10.3389/fneur.2022.925797

**Published:** 2022-07-19

**Authors:** Wupu Osimanjiang, JuliAnne E. Allgood, Rae L. Van Sandt, Daniel T. Burns, Jared S. Bushman

**Affiliations:** ^1^Division of Pharmaceutical Sciences, University of Wyoming, Laramie, WY, United States; ^2^Department of Veterinary Sciences, University of Wyoming, Laramie, WY, United States

**Keywords:** spinal cord injury, sex difference, functional outcome, lesion, immune cell recruitment

## Abstract

Spinal cord injury (SCI) is a devastating disorder, which impacts the lives of millions of people worldwide with no clinically standardized treatment. Both pro-recovery and anti-recovery factors contribute to the overall outcome after the initial SCI. Sex is emerging as an important variable, which can affect recovery post-SCI. Contusion SCI at T10 was generated in male and female rats. Open-field Basso, Beattie, Bresnahan (BBB) behavioral test, Von Frey test, and CatWalk gate analysis were performed. Histological analysis was performed at the 45-day post-SCI end point. Male/female differences in sensorimotor function recovery, lesion size, and the recruitment of immune cells to the lesion area were measured. A group of males with less severe injuries was included to compare the outcomes for severity. Our results show that both sexes with the same injury level plateaued at a similar final score for locomotor function. Males in the less severe injury group recovered faster and plateaued at a higher BBB score compared to the more severe injury group. Von Frey tests show faster recovery of sensory function in females compared to both male groups. All three groups exhibited reduced mechanical response thresholds after SCI. The lesion area was significantly larger in the male group with severe injury than in females, as well as in males of less severe injury. No significant differences in immune cell recruitment were identified when comparing the three groups. The faster sensorimotor recovery and significantly smaller lesion area in females potentially indicate that neuroprotection against the secondary injury is a likely reason for sex-dependent differences in functional outcomes after SCI.

## Introduction

Spinal cord injury (SCI) is a devastating condition, which affects more than 250,000 people in the USA, with more than 10,000 new cases each year (1, 2). SCI begins with an initial primary injury followed by a series of complex secondary pathological processes (3). The effects of the primary injury occur within minutes to hours following the initial injury while secondary injury processes proceed from immediately after injury to months after SCI (4, 5). Symptoms of SCI vary depending on the location and severity of the injury, but generally include incomplete sensory loss to complete sensorimotor deficit below the injury site, loss of bladder function, neurogenic bowl dysfunction, and neuropathic pain (6–9).

While the prevalence of SCI is higher among males, the incidence of SCI in females is increasing (10). Sex appears to be a significant factor affecting outcomes following SCI, but its extent remains unclear because the inclusion of females in clinical studies is often underpowered and the vast majority of animal studies use a single sex (11, 12). However, some indications of sexual dimorphism have emerged; improved outcomes have been observed for females 1-year post-SCI using American Spinal Injury Association (ASIA) metrics (13). Females have shown a greater susceptibility to develop nociceptive neuropathic pain (14). Female patients with SCI are less likely to experience sexual arousal than male patients with SCI, and are at elevated risk for autonomic dysreflexia, anemia, ulcers, and chronic urinary tract infections (15–18).

The mechanisms accounting for sexual dimorphism are still unclear, but some reports show some potential indications of the causes. In a study focusing on the inflammatory response comparing age and sex after SCI, significant immunological differences were discovered between males and females after SCI (19). A significantly higher amount of monocyte-derived macrophages was recruited to the injury site in female animals whereas microglia was discovered to be higher in males (19). Reactive oxygen species- (ROS-) related genes were expressed higher in females than in males whereas males expressed a higher amount of c1qa complement protein than females (19). Estrogen and progesterone have neuroprotective properties and appear to affect gliosis (20–25). Sex hormones may also contribute to the level of immune response, which may impact the progression of secondary injury (26, 27).

Experimental models have highlighted sexual dimorphisms following SCI, but not uniformly. After moderate compression SCI at T10, female mice showed evidence of improved locomotor recovery compared to males (compression level 5 g/mm^2^) (28). Female rats also exhibited greater locomotor recovery after a moderate contusion SCI at T8 (a 10-g rod dropped from a height of 12.5 mm) as measured by Basso, Beattie, Bresnahan (BBB) (females 11.39 ± 0.26 vs. males 10.27 ± 0.3 at week 4 after SCI) (29). However, other studies on mice show no differences in locomotor recovery by BMS and in reaction time to mechanical stimulation by the dynamic plantar test after severe contusion at T10 (60 kdyn) (30). Another study on rats also showed no significant sex differences in locomotor recovery using the BBB score after moderate T10 contusion SCI (a 10-g rod dropped from a height of 12.5 mm) (31).

We previously conducted a study on severe contusion at T10 in female and male rats (a 10-g rod dropped from a height of 35 mm), finding improved motor outcomes by BBB for female rats and altered metabolic parameters by sex (32). For this follow-up study, we compared outcomes for severe contusion SCI and included additional sensorimotor outcome measures and more extensive histological analysis. There were three injury groups, female and male rats with a 35-mm T10 contusion (35F and 35M, respectively), and a control group of males with a less severe contusion of 25 mm (25M) at T10. Sensorimotor outcome measures included BBB, Von Frey, and Noldus CatWalk gait analysis. When comparing the 35M and 35F groups, females not only recovered faster the sensorimotor function, but also exhibited better tissue preservation at the 45-day end point. Gate analysis has shown than females may have adapted differently to SCI than males, where differences in some aspects of baseline gait, such as crossing speed, were apparent between the sexes and complicate a direct comparison between them. Animals in the 25M group showed less severe outcomes than those in the 35M group, as expected. These data support that functional recovery and tissue preservation are influenced by both injury severity and sex.

## Materials and Methods

### Animal Care

All animals were acquired, cared for, and used in accordance with the NIH Guide for the Care and Use of Laboratory Animals and the protocols approved by the IACUC at the University of Wyoming. Rats were placed in animal rooms with temperatures between 22 and 24°C, stable humidity, 12-h day–night cycle, and free access to rodent laboratory food and water. Animals were housed in individual cages throughout the study.

### Spinal Cord Injury

Five-month-old adult male (28.1 weeks old, 500.8 ± 14.3 g) and female (26.1 weeks old, 291 ± 11.9 g) Sprague–Dawley rats were first assigned to groups based on sex, and then randomly assigned to one of the three groups: 25M group (eight animals); 35M group (eight animals); or 35F group (eight animals). Two animals in the 35M group failed to respond to antibiotics after acquiring a bladder infection and were euthanized, and the data were excluded from the analysis. SCI surgical procedures were performed as previously described (33, 34). Briefly, rats were anesthetized by inhalation of 2% isoflurane. The T10 area was palpated, and the skin was shaved and sterilized using iodine and isopropyl alcohol. The skin was incised, and the muscles around the T10 vertebrae were cleared, and a partial laminectomy was performed at T10. The spine was suspended *via* clamps near the exposed spinal cord, to ensure that the cord was parallel to the horizontal plane of the surgery table. SCI was induced using an NYU Impactor by dropping a 10 g, 1.1-mm diameter rod onto the exposed spinal cord from a height of 25 or 35 mm. The musculature and skin were sutured using 6–0 sutures. After surgery, animals received buprenorphine (NDC 42023-179-05, Par Pharmaceutical Chestnut Ridge, NY 10977, 0.05 mg/kg) two times daily for 3 days, and Baytril (Cat. No. 101-5977, Bayer Healthcare LLC, 5 mg/kg) one time daily for 7 days. The bladders were expressed two times daily until the recovery of bladder function was observed.

### Behavioral Assessment

The BBB scale was used for the functional evaluation of recovery as previously described (35, 36). Each rat was placed in a circular plastic enclosure and observed by two independent investigators. Rats were scored during a 4-min session, and the observers' scores were averaged to obtain the individual score. The scores of all animals within the group at each time point were measured to get the total group score.

The Von Frey test was used to measure the sensory response of the hind paws after SCI. The test was performed as previously described (37). Briefly, animals were individually placed on a mesh table after conditioning for 1 h. A series of Von Frey filaments (4, 6, 8, 10, 15, 26, 60, 100, 180, and 300 g) were applied perpendicularly to the hind paws. A response rate of 50% or greater, indicated by paw withdrawal, flinching, vocalization, orientation toward or biting the stimulus, or escape from the stimulus, was used as described to determine the response over 10 applications (38, 39). After testing both the sides, the hind paw responses were averaged to obtain the response threshold for that animal. The average of all animals within the group was calculated and taken as the group threshold.

### Gate Analysis

The Noldus CatWalk XT system was used to evaluate the recovery of locomotor function following SCI. The CatWalk XT system is an enclosed glass platform with openings at each end with a green light illuminating the platform (threshold 0.12) and a red light above the platform. After 3 weeks of acclimation and conditioning training, each rat voluntarily ran from one end of the platform to the other while a camera was placed at 42 cm below the platform to record foot placements. At least three compliance runs were recorded for each rat at baseline and weekly (8, 15, 21, 29, 36, and 43 days) after SCI. Upon completion of the study, each CatWalk run was auto-classified by the CatWalk software with a follow-up done by an independent investigator to correct any misidentified steps. The regularity index (%) of the hind paws, speed of crossing (s), stand duration (s), and the base of support (BOS) of the front paws were calculated using the CatWalk software after the classification was complete. Observational data, the percentage of hind limbs not dragging, the direction of hip drag relative to the CatWalk surface, plantar paw placement, and laterality of paw stepping, were also collected by an independent investigator to characterize the patterns of hind limb dragging that the CatWalk machine could not classify.

### Tissue Processing, Histology, and Immunohistochemistry

At the end point [45 days post injury (DPI)], animals were euthanized *via* perfusion with 4% paraformaldehyde (PFA) in 1× phosphate buffer solution (PBS) (Cat. No. 14200075, Life Technologies). Briefly, animals were anesthetized *via* isoflurane, and the chest cavity was opened and perfused intracardially with 60 ml of 0.9% saline followed by 30 ml of 4% PFA. A 2-cm spinal cord segment was harvested at the lesion site, and the rostral end of the tissue was labeled by cutting the segment at an angle to distinguish it from the caudal end. The tissue was then stored in 4% PFA. Before cutting, the samples were placed in 30% sucrose for 24 h, followed by optimal cutting temperature (OCT) compound (Cat. No. 625501-01, Sakura Finetek USA, Inc.) for embedding. Transverse sections of 20 μm were sliced along the entire length of the tissue from the rostral end to the caudal end using a cryostat (Leica Biosystems CM3050 S).

Hematoxylin and Eosin (H&E) staining was performed as previously described (40). Briefly, transverse sections with every 200 μm distance were washed with PBS for 5 min, before staining with hematoxylin (Cat. No. ab220365, abcam) for 45 s. After washing in tap water for 10 min, the sections were stained with Eosin Y (Cat. No. HT110116, Sigma) for 2 min. Sections were then washed in tap water for 5 min, mounted with mounting media, and cover slipped (Cat. No. F6182, Sigma). Sections were imaged with the Zeiss Axio slide scanner (Zeiss Axio Scan.Z1), and Image J FIJI software (NIH) was used to identify the lesion length and epicenter. Lesion areas within tissue sections are identified based on aberrations from unaffected tissues, such as gross discolorations, tissue fragmentation, and tissue loss. The first slice containing a lesion moving from the rostral side was identified as the rostral end of the lesion, and the last slice containing a lesion was identified as the caudal end of the lesion. Lesion length was determined by measuring the distance between the rostral and caudal ends of the lesion. The slice with the largest lesion area was determined as the lesion epicenter.

Immunohistochemistry was performed as previously described (33). Briefly, fixed sections of spinal cord tissue were washed with 1× PBS three times for 5 min each and were blocked using AB media (10% BSA, 1% normal goat serum, 0.3% Triton X-100 in 1× PBS) for 1 h. Sections were incubated with primary antibodies diluted in AB media (1% BSA, 0.1% normal goat serum, 0.3% Triton X-100 in 1× PBS) at 4°C overnight, washed three times for 5 min each, and incubated with secondary antibodies in AB media (1% BSA, 0.1% normal goat serum, 0.3% Triton X-100 in 1× PBS) for 2 h. Then, the sections were washed three times for 5 min each and stained with DAPI (Cat. No. 62248, ThermoFisher Scientific, diluted 1:1,000 in 1× PBS) for 7 min. After washing, the slides were covered with mounting media and cover slipped (Cat. No. F6182, Sigma).

For staining of astrocytes, anti-glial fibrillary acidic protein (GFAP) antibody (1:200, chicken IgY, Cat. No. AB5541, Millipore) was used, followed by Alexa Flour 647 goat anti-chicken IgY antibody (1:400, Cat. NO. A21449, ThermoFisher Scientific). For CD4 T cell staining, anti-CD4 antibody (1:200, mouse IgG2a, Cat. No. SAB4700733, Millipore) was used, followed by Alexa Flour 488 goat anti-mouse IgG antibody (1:400, Cat. No. A11001, ThermoFisher Scientific). For CD8 T cell staining, anti-CD8 antibody (1:200, rabbit IgG, Cat. No. BS-4791R, ThermoFisher Scientific) was used, followed by Alexa Flour 555 goat anti-rabbit IgG antibody (1:400, Cat. NO. A21428, ThermoFisher Scientific). For microglial cell staining, TMEM119 antibody (1:200, rabbit IgG, Cat. No. NBP2-30551, Novus Biologicals) was used, followed by Alexa Flour 488 goat anti-rabbit IgG antibody (1:400, Cat. NO. A11008, ThermoFisher Scientific). For macrophage staining, anti-CD169 antibody (1:200, mouse IgG2a, Cat. No. MA1-80164, ThermoFisher Scientific) was used, followed by Alexa Flour 555 goat anti-mouse IgG antibody (1:400, Cat. NO. A21422, ThermoFisher Scientific).

Sections were imaged on a confocal microscope (Zeiss), and Image J software (NIH) was used to measure fluorescence intensities. GFAP-stained sections were used to measure the total tissue area and lesion cavity area. The tissue was stained every 1 mm and the lesion area was measured with an error distance of ±100 μm. After measuring the lesion area with image J, the numbers were converted from pixels to micrometers and millimeters. Bright filed images were taken using a transmitted light detector (TPMT) under a confocal microscope (Zeiss).

### Assessment of Lesion Volume

The lesion area of the cross-sections was measured by GFAP staining. Total tissue volume (mm^3^), lesion volume (mm^3^), spared tissue volume (mm^3^), spared white matter volume (mm^3^), spared gray matter volume (mm^3^), and spared ventral white matter volume (mm^3^) were calculated with Cavalier's Estimator of Morphometric volume on 7 μm × 20 μm thick tissue sections spaced 1 mm apart, as previously described (41, 42).

### Statistical Analysis

Experimenters assessing sensorimotor and histological outcomes were blinded to the experimental groups during data collection. Sample size was not predetermined as repeated measures on small numbers are known to produce biologically relevant outcomes (43, 44). Microsoft Excel and SPSS software were used to perform the statistical analysis. Statistical tests are indicated in the figure legends, where *p* ≤ 0.05 was considered a significant difference. Repeated measures analysis of variance (ANOVA) was applied when comparing the means of three or more groups at multiple time points where the participants are the same in each group. For outcome measures, such as Von Frey, when all animals did not recover measurable outcomes at the same timeline, the repeated-measures Wilcoxon rank sum test was applied when at least three or more animals in a group registered a response. Bonferroni *post-hoc* analysis was conducted. Single-factor ANOVA was applied to quantifiable histological outcomes from a single time point.

## Results

### Generation of Spinal Cord Injuries and Locomotor Recovery

**Figure 1:**
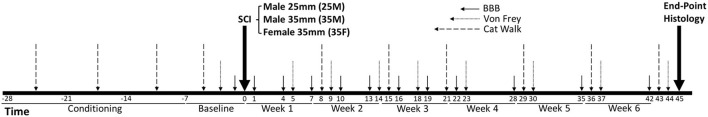
Timeline of the experiment. Horizontal axes are in days, the day of the injury is recorded as day 0, post-injury dates are in positive numbers, pre-injury dates are in negative numbers. SCI, spinal cord injury; 25M, male 25-mm injury group; 35M, male 35-mm injury group; 35F, female 35-mm injury group. *n* = 6 for the 35M group, *n* = 8 for 25M and 35F groups.

**Figure 2:**
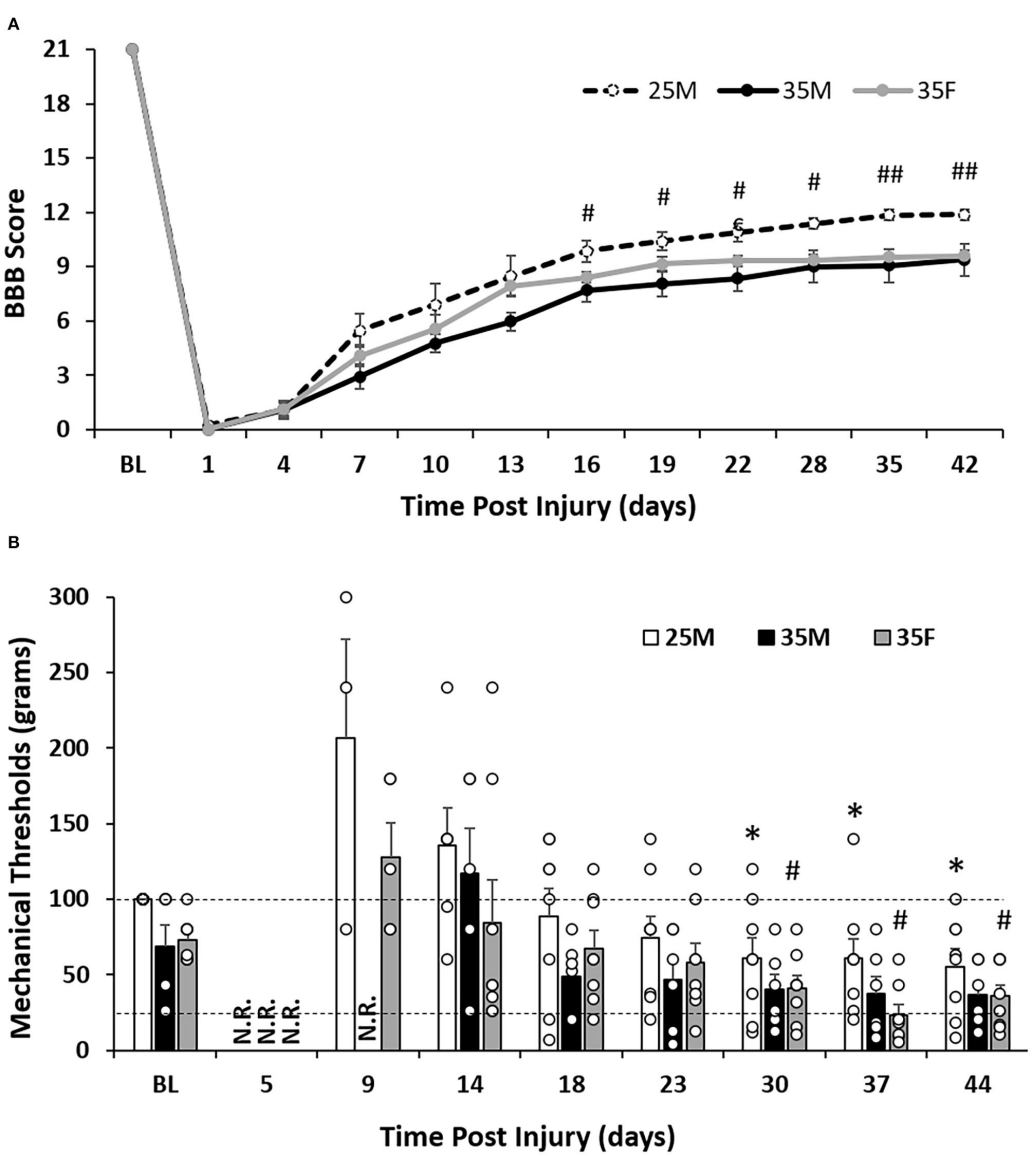
Sensorimotor functional outcomes after SCI. **(A)** Level of functional recovery over time after SCI as assessed by BBB scale scoring. Data are presented as mean ± standard error of the mean (SEM) [^#^*p* < 0.05; ^##^*p* < 0.01 between the 25M and 35M groups; repeated-measures analysis of variance (ANOVA)]. **(B)** Mechanical response thresholds assessed by the Von Frey manual test. Two dashed lines show response values of 26 and 100 g, respectively. Data are presented as mean + SEM. For timepoint baseline, 18, 23, 30, 37, and 44 DPI (*n* = 6 for the 35M group, *n* = 8 for the 25M and 35F groups); for timepoint five DPI (*n* = 2 for the 35F group, *n* = 0 for the 25M and 35M groups); for timepoint nine DPI (*n* = 5 for the 35F group, *n* = 3 for the 25M group, and *n* = 2 for the 35M group); and for timepoint 14 DPI (*n* = 8 for the 35F group, *n* = 6 for the 25M group, and *n* = 5 for the 35M group). Groups had a minimum of three rats with recorded responses (*p < 0.05 comparing the 25M group at post-injury time points with 25M baseline; ^#^*p* < 0.05 comparing the 35F group at post-injury time points with 35F baseline; repeated-measures Wilcoxon rank sum test). N.R., no record (no response to the highest filament size); BL, baseline.

Following CatWalk training and baseline data collection, contusion SCI was generated at T10 by dropping a 10-g rod from a height of 25 or 35 mm (Figure 1). BBB scores were measured one time before the injury and approximately every 3 days post-injury (Figure 1). For the 25 mm male group (25M), the BBB score fell to 0.22 ± 0.15 at one DPI, then gradually increased to 8.47 ± 1.11 at 13 DPI before plateauing at 22 DPI and ending at a BBB score of 11.88 ± 0.52 (Figure 2A). This level of functional impairment and recovery over time is consistent with previous reports (35, 45). The BBB score patterns for the 35-mm male group (35M) showed a decline to zero at one DPI, before rising to 5.96 ± 0.52 at 13 DPI, and plateauing at 22 DPI with the end point score of 9.38 ± 0.86 (Figure 2A). The 35-mm female group (35F) showed similar baseline BBB scores to the males before falling to zero at one DPI. Following this drop, 35-mm females increased to 7.91 ± 0.50 at 13 DPI before plateauing at 22 DPI with a final score of 9.59 ± 0.32 (Figure 2A).

The 35M group showed a slower recovery than the 25M group with a score of 2.92 ± 0.72 compared to the 25M group which scored 5.47 ± 0.86 at seven DPI (Figure 2A). The 25M group also plateaued at a significantly higher BBB score than the 35M group despite the scores leveling out at a similar time point post-injury. These results indicate that the severity of the injury is a determining factor of locomotor functional outcomes, which is consistent with previous research (35). The locomotor recovery, BBB score, of the 35F group plateaued at a similar level and time point with the 35M group. At the end point, both 35 mm injury groups reached scores between 9 and 10. This score indicates that the weight supported the placement of hind paws but without plantar stepping. At 13 DPI, the 35M and 35F groups showed a significant difference (*p* = 0.022) with single-factor ANOVA, close significance (*p* = 0.056) with two-way ANOVA, but no significance with repeated measures ANOVA.

### Sensory Analysis After SCI

Baseline manual Von Frey testing was performed once before the SCI, then approximately every 4 days afterward (Figure 1). At the baseline, animals from all three groups responded to one of the following three filaments, 26, 60, or 100 g, with either of their hind paws. This indicates that 26–100 g is a normal range for withdrawal threshold. Baseline recordings for the groups showed that the 35F group responded to an average filament of 72.9 ± 14.6 g, the 35M group responded an average filament of 68.7 ± 14.2, and all animals of the 25M group responded to the filament of 100 (Figure 2B).

After SCI, the 35F group regained sensory function before males (Figure 2B). At five DPI, two out of eight female rats responded to the 300-g filament, the largest filament available, whereas no animals from either 25M group or 35M group responded to any of the filaments. At nine DPI, five of the eight animals from the 35F group responded to one of the filaments (128 ± 22.5 g), three of the eight animals from the 25 M group responded to one of the filaments (206.7 ± 65.7 g), but only two of the six animals in the 35 M group responded. At 14 DPI, all eight rats from the 35F group responded to one of the filaments with the average threshold of 84.1 ± 28.7 g, whereas only six animals for the 25M group and five animals for the 35M group responded to one of the filaments with average thresholds of 135.8 ± 24.7 and 117.2 ± 29.7 g, respectively. These results indicate that female animals recovered before males, and males with less severe injury recovered sooner than more severely injured males.

Mechanical allodynia is a painful sensation that is caused by a non-painful stimulus. It is a response that is often developed in the hind paws of rats after moderate-to-severe SCI (46, 47). The mechanical response threshold for the 25M group dropped to 55.5 ± 11.5 g at 44 DPI, which is significantly lower than the baseline (Figure 2B). Although the 35M group dropped to 36.8 ± 8.4 at 44 DPI, no significant difference was seen in comparison to the baseline using the repeated measures Wilcoxon rank sum test (Figure 2B). The 35F group started to show reduced threshold at 30 DPI with 41.1 ± 8.2 g and reached the lowest threshold at 37 DPI with 23.3 ± 6.7 g (Figure 2B). These results indicate the presence of hypersensitivity in the hind paws after SCI, which may suggest the development of mechanical allodynia. The strongest example of this is in the 35F group, which reached below the normal range of 26–100 g at 37 DPI.

### Gate Analysis

Significant hind limb dragging behavior was observed in all groups for 1 week after injury and continued to persist for the 35M and 35F groups in some forms during subsequent weeks. The patterns of dragging behavior are shown in Supplementary Figure 2B. At baseline, all groups showed normal gait patterns with all hind and front limbs stepping. At 8 DPI, rats in all groups showed complete hind limb dragging (Supplementary Figure 2B part 4). At 15 DPI, new patterns of dragging emerge in most rats in each group (Supplementary Figure 2B part 2, 3, 5, and 6). Despite these new dragging patterns and data that appeared to indicate that females displayed more dragging behavior than males, there was no significant difference in the percentage of hind limbs that dragged between males and females at any time point (Supplementary Figure 2A).

As a result of over 90% of both the 35M and 35F groups maintaining the dragging behavior up to the 6-week end point, a new definition of stepping was determined. The run data were re-classified with a new definition whereby “stepping” was any motion consistent with a step, in which the hind paw started behind the hip and made one complete revolution to end in front of the hip. The paw did not have to bear any weight to be considered as a step, nor did the rats hips have to be parallel relative to the CatWalk surface. However, it was observed that most of the time, if the foot made a stepping motion, the weight was born on some portions of the hind paw.

**Figure 3:**
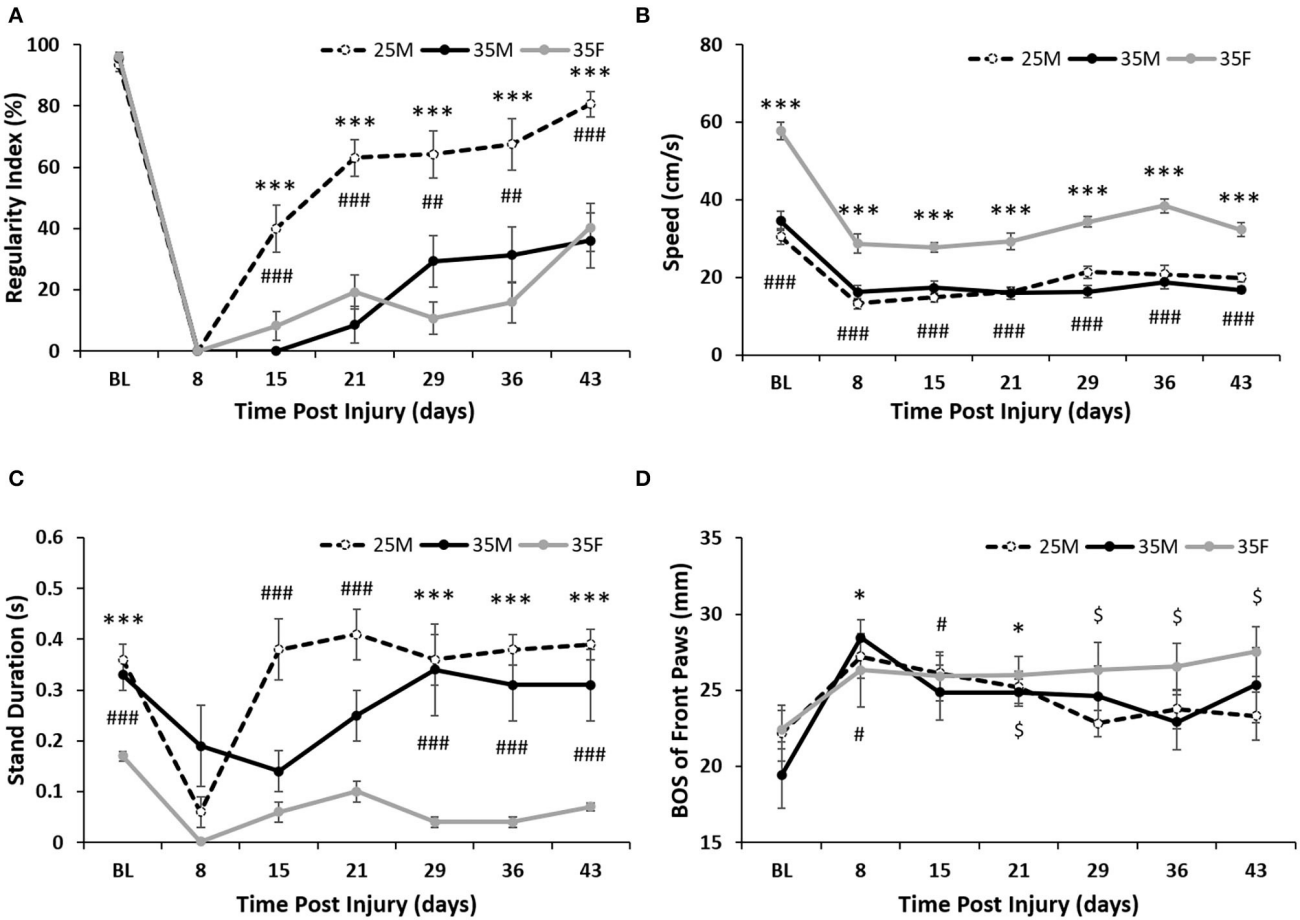
Gate analysis following SCI. **(A)** Regularity index for the hind paws as calculated by the CatWalk software. Data are presented as mean ± SEM (***p < 0.001 comparing the 25M and 35F groups; ^##^*p* < 0.01, ^###^*p* < 0.001 comparing the 25M and 35M groups; one-way repeated ANOVA with Bonferroni *post-hoc* analysis). **(B)** Average crossing speed (cm/s) as calculated by the CatWalk software. Data are presented as mean ± SEM (***p < 0.001 comparing the 35F and 25M groups; ^###^*p* < 0.001 comparing the 35F and 35M groups; one-way repeated ANOVA with Bonferroni *post-hoc* analysis). **(C)** Average stand duration for the hind paws as calculated by the CatWalk software. Data are presented as mean ± SEM (***p < 0.001 comparing the 35F and 35M groups; ^###^*p* < 0.001 comparing the 35F and 25M groups; one-way repeated ANOVA with Bonferroni *post-hoc* analysis). **(D)** Base of support (BOS) for the front paws as calculated by the CatWalk software. Data are presented as mean ± SEM (*p < 0.05 comparing the 35M group with baseline; ^#^*p* < 0.05 comparing the 25M group with baseline; ^$^*p* < 0.05 comparing the 35F group with baseline; Wilcoxon analysis).

Regularity index (%) is calculated by the CatWalk software and has the equation: Regularity index (%) = (Number of normal step sequence patterns/Total number of paw placements) × 100%. At every time point, the 25M group showed significantly fewer missteps (defined and identified by the CatWalk software) than the 35M and 35F groups, which indicates that their injury was less severe and they were able to recover before the other two groups and with more functional recovery (Figure 3A). There was no significance between the 35M and 35F groups, indicating that the severity of their injury was comparable. At 15 and 21 DPI, the 35M group (0, 8.61 ± 5.91%) showed a greater number of missteps than the 35F (8.16 ± 4.67%, 19.16 ± 5.64%) group, but this pattern switched at 29 and 36 DPI (35M 29.27 ± 8.41, 31.43 ± 9.17%, and 35F 10.71 ± 5.18, 15.96 ± 6.69%). Both 35M (36.12 ± 9.08%) and 35F (40.34 ± 7.79%) groups ended with similar regularity index percentages, which are consistent with the BBB, and showed that they were able to recover half the function of the 25M group (80% ± 4.05%).

One factor that impacted other CatWalk parameters was the speed at which females crossed the platform in comparison with males (Figure 3B). Females showed significantly faster crossing speeds at each time point than either male group, which could have affected the gait analysis post-injury. The faster crossing speed of females is due to reduced stand duration on their hind paws as compared to males (Figure 3C). The 25M group consistently showed the highest hind paw stand duration, while the 35M group showed the greatest week-to-week variability.

The dragging observed in the more severely injured groups necessitated that the level of compensation could be measured in all groups. Front paw BOS changed significantly in the female group as early as 4 weeks post-injury, whereas the base of stance did not change significantly in the 25M subjects and only changed at weeks 1 and 3 for the 35M group (26.33 ± 1.83 for 35F at week 4, 28.44 ± 1.22 and 24.85 ± 0.89 for 35M at weeks 1 and 3) (Figure 3D). From baseline (22.41 ± 1.26 cm) to 43 DPI (27.52 ± 1.64 cm), their front paw BOS increased almost 5 mm to compensate for their speed of crossing and their unique dragging patterns. Male groups did not show this level BOS accommodation, possibly indicating that they were attempting to return to normal gait with parallel hip placement and weight distributed back to their hind limbs.

The number of rats capable of having full, weight-bearing, plantar paw placement at each time point is shown in Supplementary Table 2. The 25M group showed the greatest number of rats with complete plantar paw placement at the end point (six rats), while all 35F rats demonstrated the greatest number of non-plantar paw placements at the end point (eight rats). The 35M group showed the highest dispersion of paw placement patterns at a 6-week post-SCI with three animals showing rats showing full, weight-bearing, plantar paw placement, two animals showing non-plantar, weight bearing, paw placement, and one animal showing no paw placement.

Hip orientation on the CatWalk surface as the rat traversed the platform was also observed and recorded (Supplementary Table 1). The 25M group showed the fastest return to normal stepping, with two animals returning to regular stepping by week 2 and seven animals returning to regular stepping by week 3. The 35M group had two animals return to normal stepping patterns at week 4, but most of the animals in that group maintained left hip drag with the right hind paw stepping for the remainder of the study (Supplementary Tables 1, 3). The 35F group had one hip drag at almost all time points; however, both feet started to show alternative stepping in a majority of the cases by week 3 (Supplementary Tables 1, 3). This could indicate a behavioral adaptation to compensate for the injury that female rats maintained despite being capable of making stepping motions with both hind limbs.

### Analysis of Lesion Size

**Figure 4:**
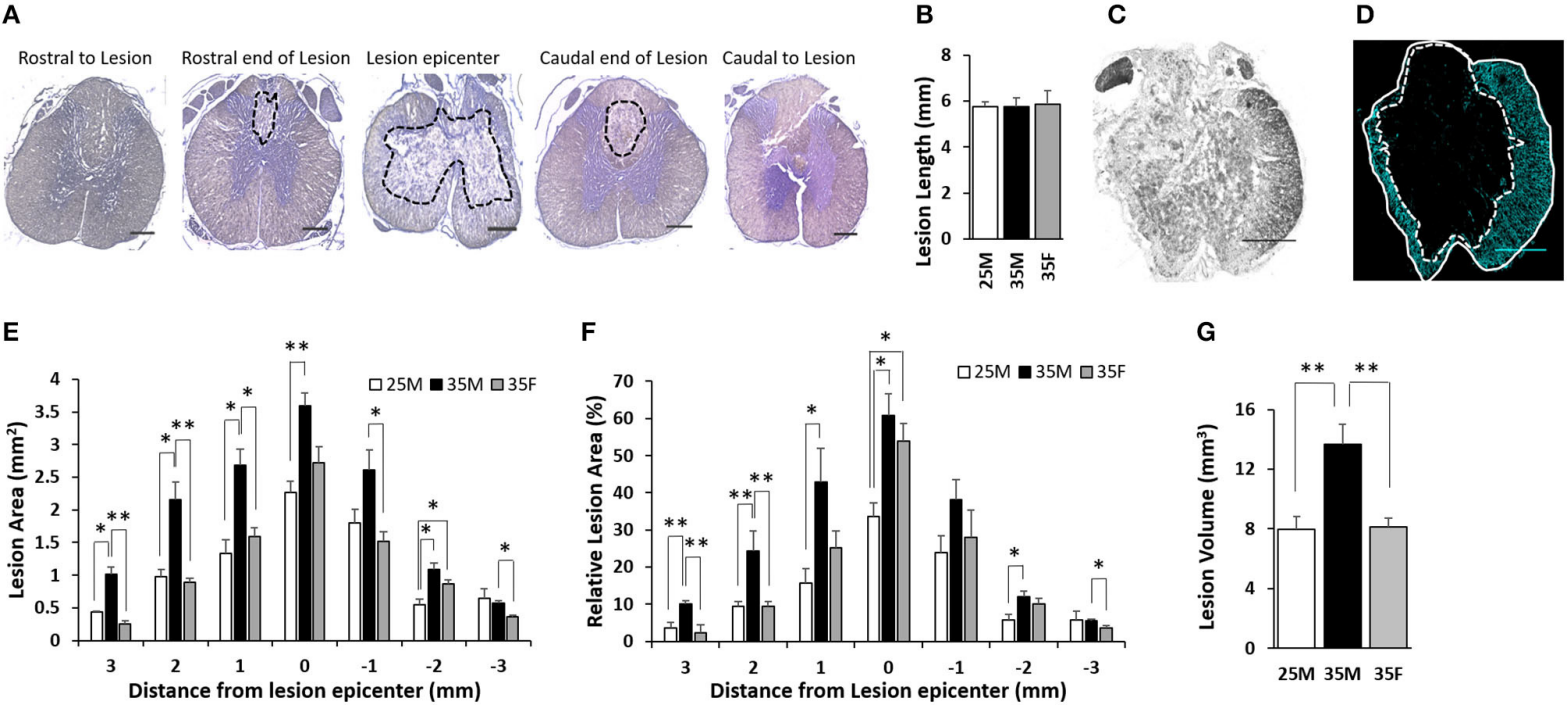
Lesion characteristics and size following SCI. **(A)** Representative images of H&E staining that show the lesion epicenter, the rostral end, and the caudal ends of the lesion. Lesion areas are circled in black dashed lines. The rostral end of the lesion and the caudal end of the lesion are each 3 mm rostral or caudal, respectively, from the lesion epicenter on average. Rostral to lesion and caudal to lesion are 400 μm rostral and caudal, respectively, from the rostral end of the lesion and the caudal end of the lesion. **(B)** Quantification of the longitudinal length of the lesion. Data are presented as mean + SEM. **(C)** Bright field image of the lesion epicenter. **(D)** Fluorescent image of the lesion epicenter labeled for anti-glial fibrillary acidic protein (GFAP). Total tissue is circled in white line, and the lesion core is circled in white dashed line. Please see Supplementary Figure 4 for representative images of all groups at all distances. **(E)** Quantification of absolute lesion areas at various distances from the lesion epicenter. The lesion epicenter is recorded as 0, the distances rostral to the epicenter are in positive numbers, and the distances caudal to the epicenter are in negative numbers. Data are presented as mean + SEM (*p < 0.05, **p < 0.01; single-factor ANOVA). **(F)** Quantification of relative lesion areas at various distances from the lesion epicenter. Data are presented as mean + SEM (*p < 0.05, **p < 0.01; single-factor ANOVA). **(G)** Measured lesion volume. Data are presented as mean + SEM (**p < 0.01; single-factor ANOVA). Scale bar shows 500 μm.

The rostral end of the lesion, lesion epicenter, and the caudal end of the lesion were identified using H&E images (Figure 4A). The lesion longitudinal length for the three groups was 5.58 ± 0.22, 5.77 ± 0.38, and 5.85 ± 0.62 mm for the 25M, 35M, and 35F groups, respectively (Figure 4B). The lesion area was measured using GFAP images (Figure 4D). Figure 4C shows a bright field image of the lesion epicenter. Total tissue area and lesion area were quantified at a distance of 0, 1, 2, and 3 mm away from the lesion epicenter in both directions (Supplementary Figure 1; Figure 4E). The Relative lesion area was measured by dividing the lesion area by the total area and then multiplying by 100% (Figure 4F). At the lesion epicenter, the total tissue area was 7.18 ± 0.59, 6.08 ± 0.36, and 4.97 ± 0.28 mm^2^ for the 25M, 35M, and 35F groups, respectively. At 3 mm rostral to the lesion epicenter, the total tissue area was 11.9 ± 0.37, 10.8 ± 0.63, and 10.08 ± 0.32 mm^2^ for the 25M, 35M, and 35F groups, respectively. At 3 mm caudal to the lesion epicenter, the total tissue area was 11.79 ± 0.28, 10.1 ± 0.33, and 9.87 ± 0.28 mm^2^ for the 25M, 35M, and 35F groups, respectively. All three groups exhibited a significantly reduced total tissue area at the lesion epicenter, which indicates the compression of the spinal cord tissue after SCI (Supplementary Figure 1). Total tissue volume was measured using Cavalier's Estimator of Morphometric volume, and the results showed a significantly larger volume for the 25M group compared to the 35M and 35F groups, whereas two 35-mm injury groups did not display a significant difference (Supplementary Figure 5A).

At the lesion epicenter, the absolute lesion areas of the 25M, 35M, and 35F groups were 2.26 ± 0.21, 3.6 ± 0.24, and 2.72 ± 0.31 mm^2^, respectively (Figure 4E). All of these constituted 33.6 ± 3.8, 60.8 ± 5.8, and 54 ± 4.6% of the total tissue area at the lesion epicenter (Figure 4F). There is no significant difference between the 35M and 35F groups; however, the 25M group is significantly <35M group. This suggests that the lesion severity was similar for the two 35-mm groups, but less severe for the 25-mm male group. The lesion area gradually decreased for all three groups that are moving rostrally and caudally (Figures 4E,F). The lesion areas 1 mm rostral and caudal to the lesion epicenter are 1.33 ± 0.21 and 1.8 ± 0.22 mm^2^, 2.69 ± 0.24 and 2.61 ± 0.31 mm^2^, and 1.59 ± 0.14 and 1.52 ± 0.14 mm^2^ for the 25M, 35M, and 35F groups, respectively. These areas constitute 15.7 ± 3.8 and 23.9 ± 4.5%, 42.9 ± 9 and 38.1 ± 5.5%, and 25.3 ± 4.5 and 28.1 ± 7.2% of the total tissue area. At this distance, the 35F group showed a significantly smaller absolute lesion area compared to the 35M group, but the significance is not retained when comparing relative tissue area. The 25M group displayed a significantly smaller lesion in contrast to the 35M group at 1 mm on the rostral side, but not on the caudal side. At 2 mm rostral and caudal to the lesion epicenter, the lesion areas are 0.98 ± 0.1 and 0.54 ± 0.09 mm^2^, 2.15 ± 0.27 and 1.09 ± 0.09 mm^2^, and 0.89 ± 0.07 and 0.86 ± 0.07 mm^2^ for the 25M, 35M, and 35F groups, respectively, which constitute 9.5 ± 1.2 and 5.7 ± 1.7%, 24.4 ± 5.4 and 12.1 ± 1.5%, and 9.5 ± 1.2 and 10.1 ± 1.6%. At this distance, the 35F group exhibited smaller lesion compared to the 35M group only on the rostral side, not on the caudal side. The 25M group displayed a significantly smaller lesion area than the 35M group on both sides. At 3 mm rostral and caudal to the lesion epicenter, the lesion areas were 0.44 ± 0.01 and 0.64 ± 0.15 mm^2^, 1.01 ± 0.12 and 0.57 ± 0.04 mm^2^, and 0.25 ± 0.06 and 0.36 ± 0.03 mm^2^ for the 25M group, 35M group, and 35F group, respectively, which constituted 3.7 ± 1.4 and 5.8 ± 2.3%, 10.1 ± 0.8 and 5.6 ± 0.4%, and 2.4 ± 2.1 and 3.7 ± 0.6%. At this distance, the 35F group exhibited a smaller lesion compared to the 35M group only at both sides. The 25M group displayed a significantly smaller lesion than the 35M group only at the rostral side.

Total lesion volume (Figure 4G) and multiple measurements of spared tissue (Supplementary Figures 5B–E) were calculated. The results showed a significantly larger lesion volume for the 35M group compared to the 35F and 25M groups, whereas the 35F and 25M groups had comparable lesion volumes (Figure 4G). These results are consistent with lesion area measurements (Figures 4E,F). Total spared tissue, spared white matter, and spared ventral white matter volumes exhibited similar outcomes. The 25M group displayed larger volumes than the two 35-mm groups on these measurements (Supplementary Figures 5B–D), whereas the two 35-mm injury groups did not show a significant difference. Interestingly, the total spared gray matter volume was shown to be significantly smaller for the 35M group compared to the 25M and 35F groups (Supplementary Figure 5E), which indicates the impact of sex and injury severity on tissue sparing.

The 25M group displayed a significantly smaller lesion area at the lesion epicenter than the 35M group, which indicates a clear difference in the primary injury severity. This result is consistent with the behavioral outcomes. The lesion area at the epicenter was comparable for the 35M group and the 35F group, which suggests that the primary injury severity was similar for these two groups. Away from the lesion epicenter, both the 25M and 35F groups exhibited a significantly smaller lesion area compared to the 35M group, suggesting differences in the progression of secondary injury.

### Analysis of Astrogliosis and Cellular Infiltration

**Figure 5:**
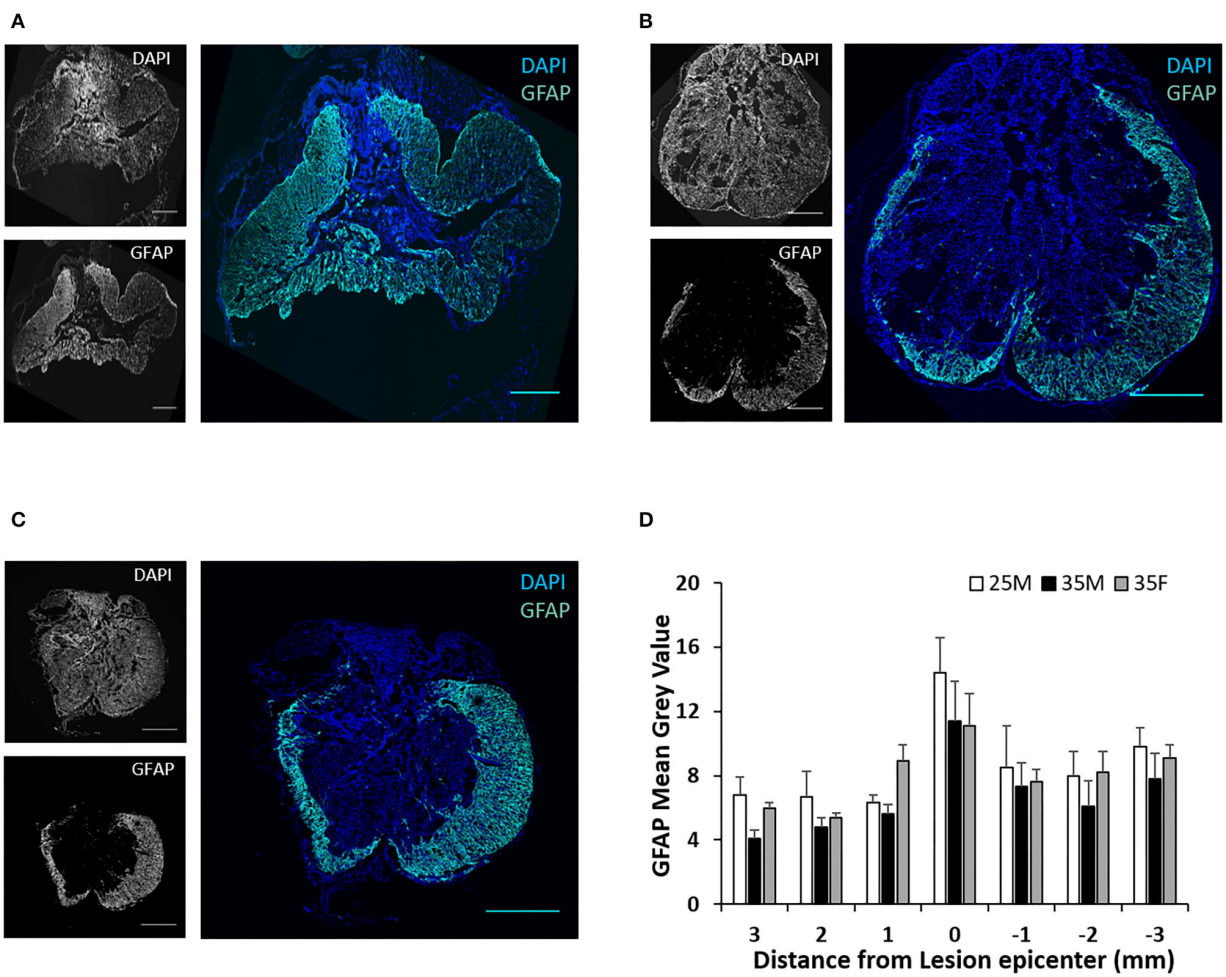
Astroglial scar at the lesion epicenter following SCI. **(A–C)** Fluorescence images of the lesion epicenter of 25M **(A)**, 35M **(B)**, and 35F **(C)** groups, DAPI (blue), GFAP (teal). **(D)** Quantification of fluorescence intensities of GFAP at various distances from the lesion epicenter. Data are presented as mean + SEM. Scale bar shows 500 μm.

Anti-GFAP antibody was used to label reactive astrocytes (Figures 5A–C). A mature SCI lesion can be divided into three tissue compartments, a central fibrotic scar lesion core, an astroglial scar border, and a surrounding zone of viable neural tissue that is spared and functional but with reactive glia (48). As shown in Figures 5A–C, the astroglial scar border surrounded the lesion core. Quantification of fluorescence intensities for GFAP exhibit reduced mean gray values away from the lesion epicenter (Figure 5D).These results are consistent with previous reports (49). However, sex or injury severity did not affect astrogliosis.

**Figure 6:**
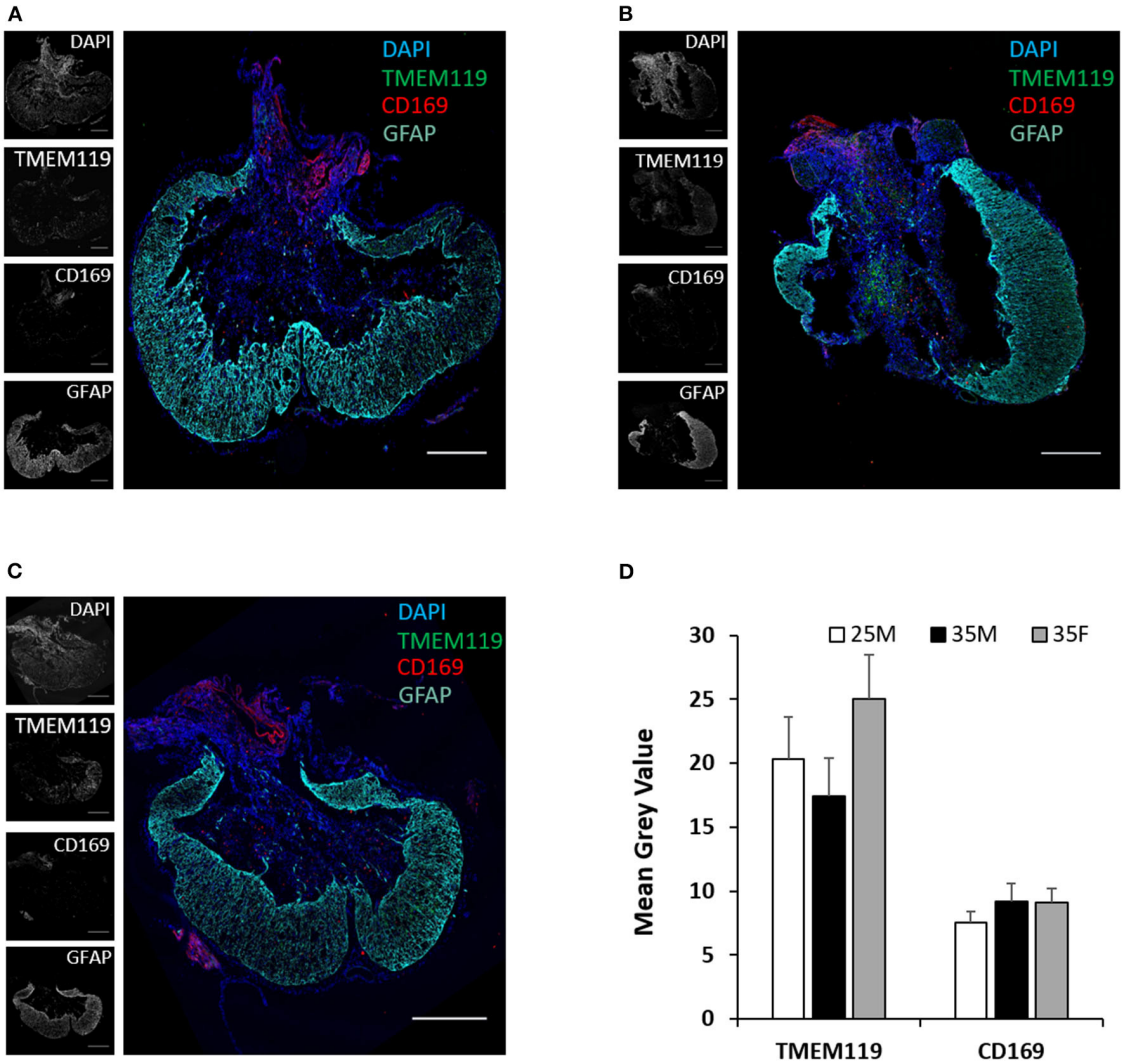
Microglia and macrophages at the lesion epicenter following SCI. **(A–C)** Fluorescence images of the lesion epicenter of 25M **(A)**, 35M **(B)**, and 35F **(C)** groups, DAPI (blue), TMEM119 (green), CD169 (red), and GFAP (teal). **(D)** Quantification of fluorescence intensities of TMEM119 and CD169 at the lesion epicenter. Data are presented as mean + SEM. Scale bar shows 500 μm.

**Figure 7:**
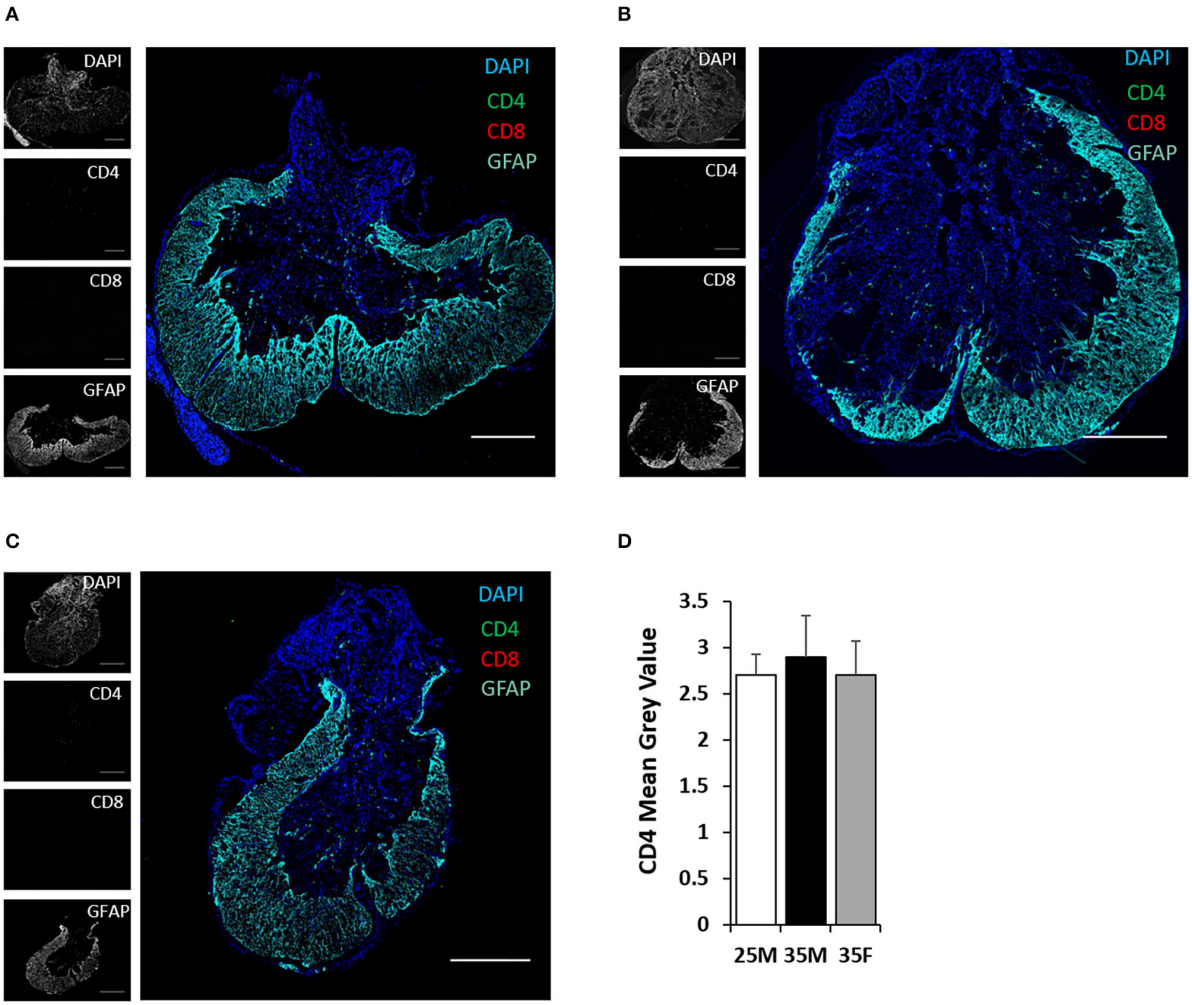
CD4 and CD8 T cells at the lesion epicenter following SCI. **(A–C)** Fluorescent images of the lesion epicenter of 25M **(A)**, 35M **(B)**, and 35F **(C)** groups, DAPI (blue), CD4 (green), CD8 (red), and GFAP (teal). **(D)** Quantification of fluorescence intensities of CD4 at the lesion epicenter. Data are presented as mean + SEM. Scale bar shows 500 μm.

TMEM119 and CD169 antibodies are more specific for microglia and blood-derived macrophages, respectively (50, 51). TMEM119 and CD169 staining at the lesion epicenter exhibits the presence of both cell types (Figures 6A–C). Microglial cells are present both in the lesion cavity and in the glial scar surrounding the lesion cavity (Figures 6A–C). CD169-positive macrophages are primarily concentrated on the dorsal side of the lesion cavity (Figures 6A–C). The quantified mean gray values of TMEM119 and CD169 show no significant differences between the groups (Figure 6D). CD4 T cells were identified at the lesion site, but there was no evidence of CD8 T cells (Figures 7A–C). Quantification of fluorescence intensities showed no significant differences between the groups (Figure 7D). These results indicate that blood-derived macrophages and CD4 T cells infiltrated the lesion after SCI and are present even at 45 DPI (Figures 6A, 7).

## Discussion

Sex is emerging as a variable, which affects functional outcomes after SCI. Although somewhat controversial, female animals can exhibit better locomotor recovery and better tissue preservation compared to male animals after SCI (27–30). In a study of moderate compression SCI performed at T10 in mice, females exhibited a higher open field BBB test score immediately after SCI and this trend continued until the end point of the study, which was 14 days post SCI (28). In a contusion SCI study at T10 in mice, no significant differences in locomotor and sensory outcomes were identified (30). In another contusion SCI study at T9 in mice, no significant differences in locomotor and sensory outcomes, as well as in the spared tissue area, were seen when comparing sex (52). After a moderate contusion SCI, only male rats developed mechanical allodynia, but not female animals (53). Regarding thermal pain, although animals of both sexes developed thermal hyperalgesia after SCI, no significant differences were identified when comparing sex (53, 54). In two different studies of contusion SCI at T8 on rats, females show a significantly higher BBB score after a 3-week post-SCI compared to males although no significant differences were discovered in the acute phase of SCI (27, 29). The different outcomes seen in these previous reports could be due to their differences in model animals, injury models, SCI location and severity, and sample sizes. In a previous pilot study reported by our group, we compared cardiometabolic and BBB outcomes in adult male and female rats and observed differences in the BBB and various cardiometabolic effects according to sex after severe contusion SCI (32).

The primary advancement of this study is to reinforce and expand the known behavioral outcomes and sex-different histological characteristics of SCI. The present study contributes to the field by including a combination of BBB, Von Frey, and CatWalk behavioral tests along with a detailed analysis of lesion size *via* histology in the same animals. Regarding locomotor function, although the BBB test shows a tendency of a more rapid recovery timeline for females compared to males with SCI, the significance was not consistent across different statistical analyses (Figure 2A). Farooque et al. performed compression injury in mice at T10 and found that female animals had a significantly better BBB score at one DPI, and females recovered better than males throughout the 14-day study (28). Hauben et al. (27) and Datto et al. (29) both performed contusion SCI at T8, and they found better recovery of females than males at later time points in their studies. However, a similar study performed by Walker et al. in Harlan rats showed no significant locomotor difference when comparing the sexes (31). The Von Frey assessment of sensory function in this study also suggest a more rapid recovery timeline in females than in males (Figure 2B). Although Fukutoku et al. also tested the mechanical reaction using the Dynamic Plantar test, they performed their tests in every 2 weeks beginning 14 days after SCI (30). The 25M and 35F groups exhibited hypersensitivity with potential development of mechanical allodynia at later time points after SCI (Figure 2B). This is consistent with previous reports that moderate and severe SCI causes the development of mechanical allodynia in rats, which was performed predominantly in female rats (146 female rats and 10 male rats were randomly placed into nine groups) (55).

Regarding histological outcomes, lesion analysis displayed a similar lesion area at the epicenter (Figures 4E,F), which suggest similar severity of the primary injury for both males and females. However, being away from the lesion epicenter, females showed reduced lesion area and estimated lesion volume compared to males of the same injury (Figure 4G). Quantification of spared tissue showed differences between 25M and both 35M and 35F groups, but no differences in gross spared tissue between 35M and 35F (Supplementary Figure 5B). However, female animals showed a greater amount of spared gray matter than males of the same injury (Supplementary Figure 5E). This outcome indicates potential secondary injury differences between males and females. Stewart et al. studied inflammatory profiles in the acute phase of SCI, and they found that C1qa complement expression is significantly increased in males whereas ROS-related genes increased in expression in females, which may lead to the divergence of SCI secondary injury toward a more complement-driven response in males and a more ROS-dependent response in females (19). Although the exact mechanisms remain unknown, this difference in secondary injury may have contributed to sex-dependent differences in functional outcomes after SCI (56, 57).

Although females recruited more macrophages and less microglia than males in the acute phase of SCI (19), this sex dependency tends to be eliminated in chronic SCI (19, 31). Our results showed no sex differences in astrogliosis, microglial reactivity, or macrophage infiltration at the 45-day endpoint, which is consistent with previous reports (31). Previous studies reported that T-cell infiltration at the lesion site peaked at seven DPI and started to decline between 1 and 2 weeks post-SCI, and these T cells observed at the injury site were predominantly CD4 T cells (58). We did not detect any CD8 T cells at the lesion epicenter in any group at the 45-day endpoint, and no significant differences of CD4 T cells in between the groups were identified (Figure 7). However, it is possible that such differences may occur at previous time points when differences in BBB and Von Frey between the sexes are developing. Many aspects of immunology diverge by sex in the absence of SCI (59–61), and there are some indications that differences in the peripheral immune response to SCI by sex contribute to outcomes (27, 62, 63). More studies on this as a potential factor contributing to sexual dimorphism in SCI are warranted.

A secondary advance of our study is the divergence between the sexes for kinetic gait analysis assessed by CatWalk. An emerging theme to assess the functional recovery after SCI is to combine BBB open field scoring with multiple CatWalk parameters (64, 65). In severe SCI, the inability to produce weight bearing steps will not allow a meaningful interpretation of CatWalk results (66). In place of weight bearing hindlimb stepping, quadrupedal animals can compensate by dragging the hind limbs and hips and rely on front legs for locomotion (67–69). In this study, females compensated for severe SCI differently from males by increasing their BOS in the front paws significantly compared to baseline by a 4-week post-SCI (Figure 3D). Observational data suggest that females also compensated for severe SCI differently from males by rotating their hips and using the wall of the CatWalk runway to push off their hindlimbs (Supplementary Figure 2). While all eight of the females rotated their hips and used the wall of the CatWalk runway, only two of six males in the 35M group used this strategy for some of their CatWalk runs. A majority of the 35F group also showed a persistent preference for dragging their left hip on the CatWalk surface despite being able to step with one or both hind paws (Supplementary Tables 1, 3). These compensation patterns after SCI have not been studied to this point, but future studies utilizing a quantitative evaluation technique may allow for a more complete characterization of the recovery process. Specifically, defining these dragging patterns and designing a way to reproducibly measure them would allow the ability to retain and analyze all post-injury runs, allowing for an analysis of the complete gait recovery process.

In addition to hindlimb dragging, significant differences in CatWalk crossing speeds of females and males limit the assessment of recovery and complicates the comparison between the sexes. Many of the CatWalk parameters using speed as a calculation factor were skewed in the female group, indicating greater recovery. Commonly reported CatWalk parameters in SCI include maximum contact in (%), stand index, and phase dispersion. Maximum contact in (%) uses the time in seconds that the paw makes maximum contact with the plate relative to the paw standing time. This is heavily impacted by crossing speeds because maximum paw contact will occur later for faster animals than for slower animals. Stand index and phase dispersions are also heavily influenced by crossing speed because both use maximum contact for calculation. As a result, crossing speed is a confounding variable in this study that hindered CatWalk interpretation for some parameters.

Differences in crossing speed may be due to a number of possible causes, including sexual dimorphisms, such as body weight, bone biomechanics, behavioral adaptations to injury, or mood changes after injury (70–72). Previous research on SCI in mice suggests that, after injury, males travel less distance with less speed and show a higher incidence of depression (73). Stand duration data indicate that males stand for a significantly longer time than females and the authors suggested that sexual dimorphism exists in mood responses to injury as stand duration is a measurable way to diagnose depression in rodents (Figure 3C) (74–76). The animal strain also be a factor, as some aspects of gait have also been found to differ by sex in some strains of mice without SCI (77). Datto et al. show differences in CatWalk by sex in response to SCI in rats, but not at baseline using Fisher rats whereas the present study was performed with Sprague–Dawley rats (29).

The inclusion of the 25M group provided a useful comparison between the groups to determine which outcome measures were most strongly influenced by injury severity vs. sex. Sex was the primary distinguishing characteristic between the 25M, 35M, and 35F groups for BOS (Figure 3D), lesion size/tissue sparing (Figure 4), stand duration, and speed (Figures 3B,C) whereas differences in the regularity index were more distinguishable by injury severity (Figure 3A). Von Frey and BBB showed indications of being influenced by both injury severity and sex, but severity created the largest differences between the groups (Figure 2). The 25M female group who has provided additional insights was not included in this study.

Significant dragging of hind limbs and alternative stepping observed in CatWalk appear to contrast with BBB observational scoring for the more severely injured animals. For the BBB score, scores of nine or above are indicative of plantar stepping and weight bearing steps. Limited traditional weight-bearing stepping was observed for BBB, but was not evident in CatWalk (Figure 2A; Supplementary Figure 2A). This divergence may be attributable to differences in these behavioral assessments and how they are recorded. Open-field BBB scoring monitors animals for several minutes and scores the animal for the highest scoring movement observed during the entire period (35). CatWalk is a task-oriented training exercise with the average data that detects where pressure exerted on the walkway can be visualized with light intensity to confirm plantar stepping and toe separation. Several runs are used to quantify the animal's behavior. Baseline sex differences exert a stronger influence on CatWalk, where the BOS also suggests that female animals adapted differently to this outcome measure relative to male animals of any injury severity.

In summary, this study assessed behavioral outcomes and histological characteristics comparing the sexes and two male groups with different injury severity. Behavioral outcomes include locomotor function, sensory function to mechanical stimulation, and compensation techniques to severe injury. Histological characteristics include lesion size at the epicenter and away from the epicenter, astrogliosis and microglial activation, and the recruitment of macrophages and T cells at 45 DPI post-SCI. Our results on sensorimotor function and lesion size exhibited some sex-dependent differences, which may give females a slight advantage for SCI recovery. The addition of a less severely injured group gave us the opportunity to compare our model and outcomes with previous reports. This moderate injury group exhibited better motor function and tissue preservation, as expected. Although the underlying mechanism of the sex-dependent differences remains unknown, their differences in the secondary injury may have contributed to it. Future studies may need to analyze differences in secondary injury progression to uncover the mechanisms of these sex-dependent outcomes after SCI.

## Data Availability Statement

The original contributions presented in the study are included in the article/Supplementary Material, further inquiries can be directed to the corresponding author/s.

## Ethics Statement

The animal study was reviewed and approved by IACUC at the University of Wyoming.

## Author Contributions

WO performed majority of the research, analyzed data, and wrote and edited the manuscript. JA performed research and analysis for CatWalk study, analyzed data, and wrote and edited the manuscript. DB helped to design the study, performed animal care, collected CatWalk data, and analyzed data. RV performed analysis for CatWalk study, analyzed data, and wrote and edited the manuscript. JB devised experiments, provided all the supplies and lab space, analyzed data, performed writing, and editing of the manuscript. All authors contributed to the article and approved the submitted version.

## Funding

This work was supported by US Department of Defense grant W81XWH-17-1-0402, the University of Wyoming Sensory Biology COBRE under National Institutes of Health (NIH) Award Number 5P20GM121310, and the National Institute of General Medical Sciences of the NIH under the Award Number P20GM103432.

## Author Disclaimer

The contents are solely the responsibility of the authors and do not necessarily represent the official views of the US Department of Defense, NIH, or the University of Wyoming.

## Conflict of Interest

The authors declare that the research was conducted in the absence of any commercial or financial relationships that could be construed as a potential conflict of interest.

## Publisher's Note

All claims expressed in this article are solely those of the authors and do not necessarily represent those of their affiliated organizations, or those of the publisher, the editors and the reviewers. Any product that may be evaluated in this article, or claim that may be made by its manufacturer, is not guaranteed or endorsed by the publisher.

## Supplementary Material

Supplementary Figure 1 Total tissue areas at various distances from the lesion epicenter. Data are presented as mean + standard error of the mean (SEM) [^*^*p* < 0.05; ^##^*p* < 0.01; ^###^*p* < 0.001; single-factor analysis of variance (ANOVA)].

Supplementary Figure 2 Hind paw placement and dragging patterns following spinal cord injury (SCI). **(A)** The average percentage of hind paws that produce a partial or full stepping motion. Data are presented as mean + SEM (^#^*p* < 0.05, one-way repeated measures ANOVA and Bonferroni *post hoc* analysis). **(B)** Schematics of the hindlimb dragging patterns observed in CatWalk runs. (1) Pre-injury gait. (2) One foot dragging, left or right, while the other foot performs stepping motion with hips parallel to the CatWalk surface. (3) Both feet move in a stepping motion, but either the left or right hip is dragging on the CatWalk surface and bearing most of the weight. (4) Both feet dragging with the hips parallel to the CatWalk surface. (5) One foot dragging while the other foot moves in a stepping motion, but the right or left hip is on the CatWalk surface and bearing most of the weight. (6) Both hind limbs are dragging with one hip on the CatWalk surface. Images of dragging patterns were produced using the Biorender software (biorender.com).

Supplementary Figure 3 Representative images of rat footprints. **(A,C,E)** Footprints before SCI for 25M **(A)**, 35M **(C)**, and 35F **(E)**, respectively. **(B, D, F)** Footprints of the same animals [25M **(B)**, 35M **(D)**, and 35F **(F)**] at 6-week post-SCI.

Supplementary Figure 4 Representative images of anti-glial fibrillary acidic protein (GFAP) staining at variance distances for all three groups. Scale bar shows 500 μm.

Supplementary Figure 5 Volume of the lesion and spared tissue. **(A)** Total tissue volume of analyzed 6-mm spinal cord tissue segment. **(B)** Volume of the total spared tissue. **(C)** Volume of the total spared white matter. **(D)** Volume of the total spared ventral white matter. **(E)** Volume of the total spared gray matter. Data are presented as mean + SEM (^*^*p* < 0.05; ^**^*p* < 0.01; single-factor ANOVA).

Supplementary Table 1 Direction of hip dragging relative to CatWalk surface.

Supplementary Table 2 Plantar paw placement patterns observed on the CatWalk surface.

Supplementary Table 3 Laterality patterns of paw stepping observed on the CatWalk surface. RH, right hind paw; LH, left hind paw.
