# Supplementary figures and images for: Sexual Dimorphism in Lesion Size and Sensorimotor Responses Following Spinal Cord Injury
Source: Front Neurol. 2022 Jul 19;13:925797. doi: 10.3389/fneur.2022.925797 (PMC10041393; doi:10.3389/fneur.2022.925797)

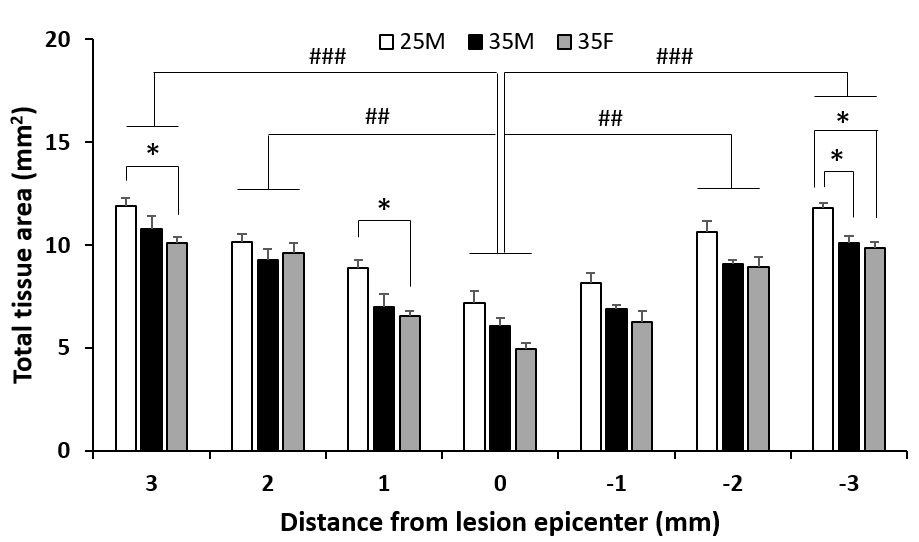

Supplement: Supplementary Figure 1 — Total tissue areas at various distances from the lesion epicenter. Data are presented as mean + standard error of the mean (SEM) [*p < 0.05; ##p < 0.01; ###p < 0.001; single-factor analysis of variance (ANOVA)]. [file Image_1.TIF]

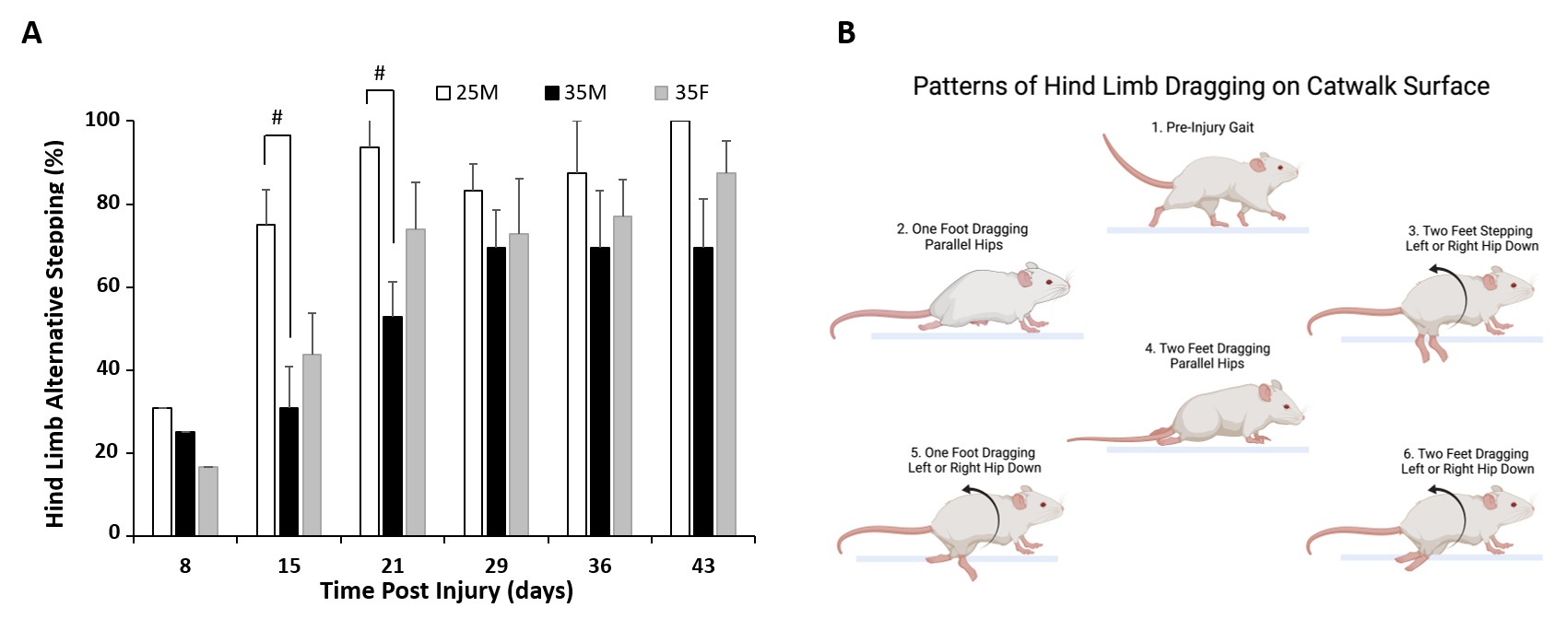

Supplement: Supplementary Figure 2 — Hind paw placement and dragging patterns following spinal cord injury (SCI). (A) The average percentage of hind paws that produce a partial or full stepping motion. Data are presented as mean + SEM (#p < 0.05, one-way repeated measures ANOVA and Bonferroni post hoc analysis). (B) Schematics of the hindlimb dragging patterns observed in CatWalk runs. (1) Pre-injury gait. (2) One foot dragging, left or right, while the other foot performs stepping motion with hips parallel to the CatWalk surface. (3) Both feet move in a stepping motion, but either the left or right hip is dragging on the CatWalk surface and bearing most of the weight. (4) Both feet dragging with the hips parallel to the CatWalk surface. (5) One foot dragging while the other foot moves in a stepping motion, but the right or left hip is on the CatWalk surface and bearing most of the weight. (6) Both hind limbs are dragging with one hip on the CatWalk surface. Images of dragging patterns were produced using the Biorender software (biorender.com). [file Image_2.TIF]

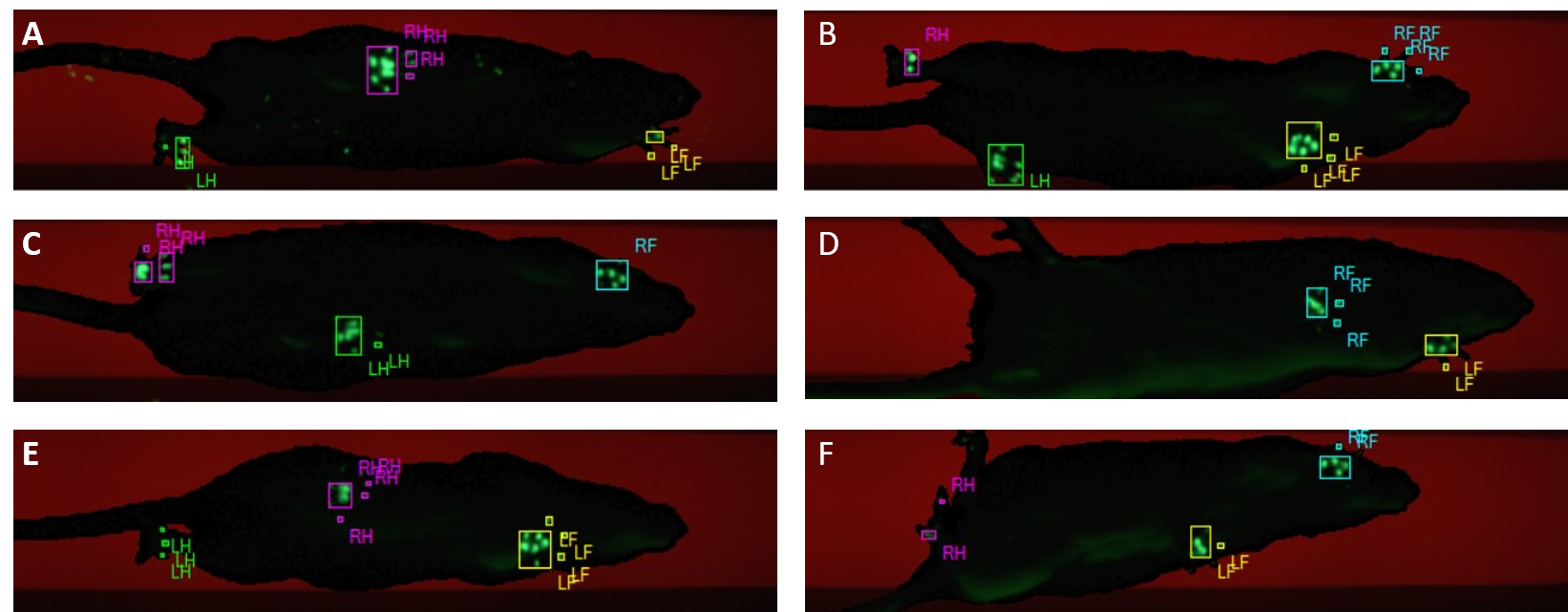

Supplement: Supplementary Figure 3 — Representative images of rat footprints. (A,C,E) Footprints before SCI for 25M (A), 35M (C), and 35F (E), respectively. (B, D, F) Footprints of the same animals [25M (B), 35M (D), and 35F (F)] at 6-week post-SCI. [file Image_3.TIF]

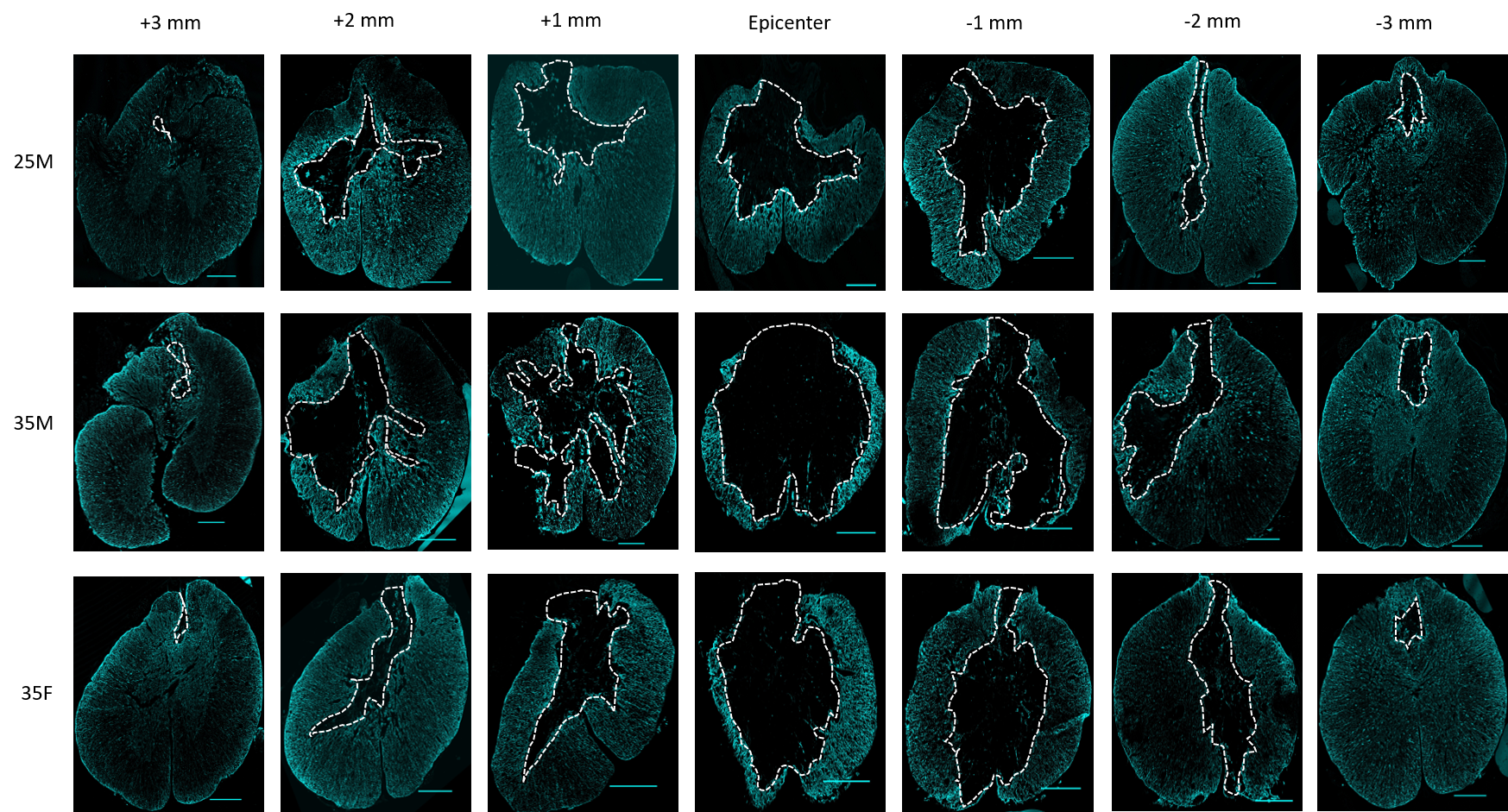

Supplement: Supplementary Figure 4 — Representative images of anti-glial fibrillary acidic protein (GFAP) staining at variance distances for all three groups. Scale bar shows 500 μm. [file Image_4.TIF]

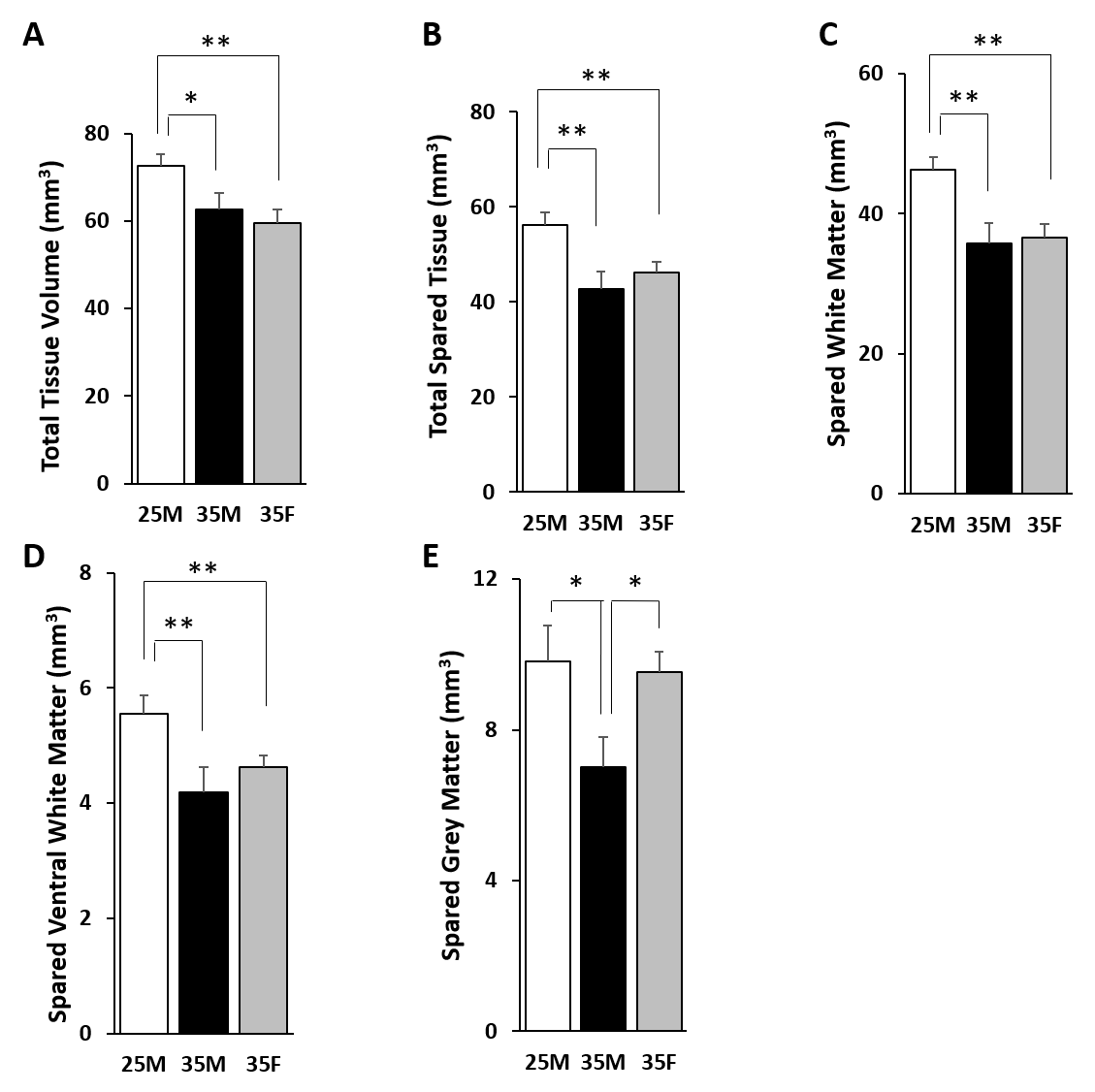

Supplement: Supplementary Figure 5 — Volume of the lesion and spared tissue. (A) Total tissue volume of analyzed 6-mm spinal cord tissue segment. (B) Volume of the total spared tissue. (C) Volume of the total spared white matter. (D) Volume of the total spared ventral white matter. (E) Volume of the total spared gray matter. Data are presented as mean + SEM (*p < 0.05; **p < 0.01; single-factor ANOVA). [file Image_5.TIF]

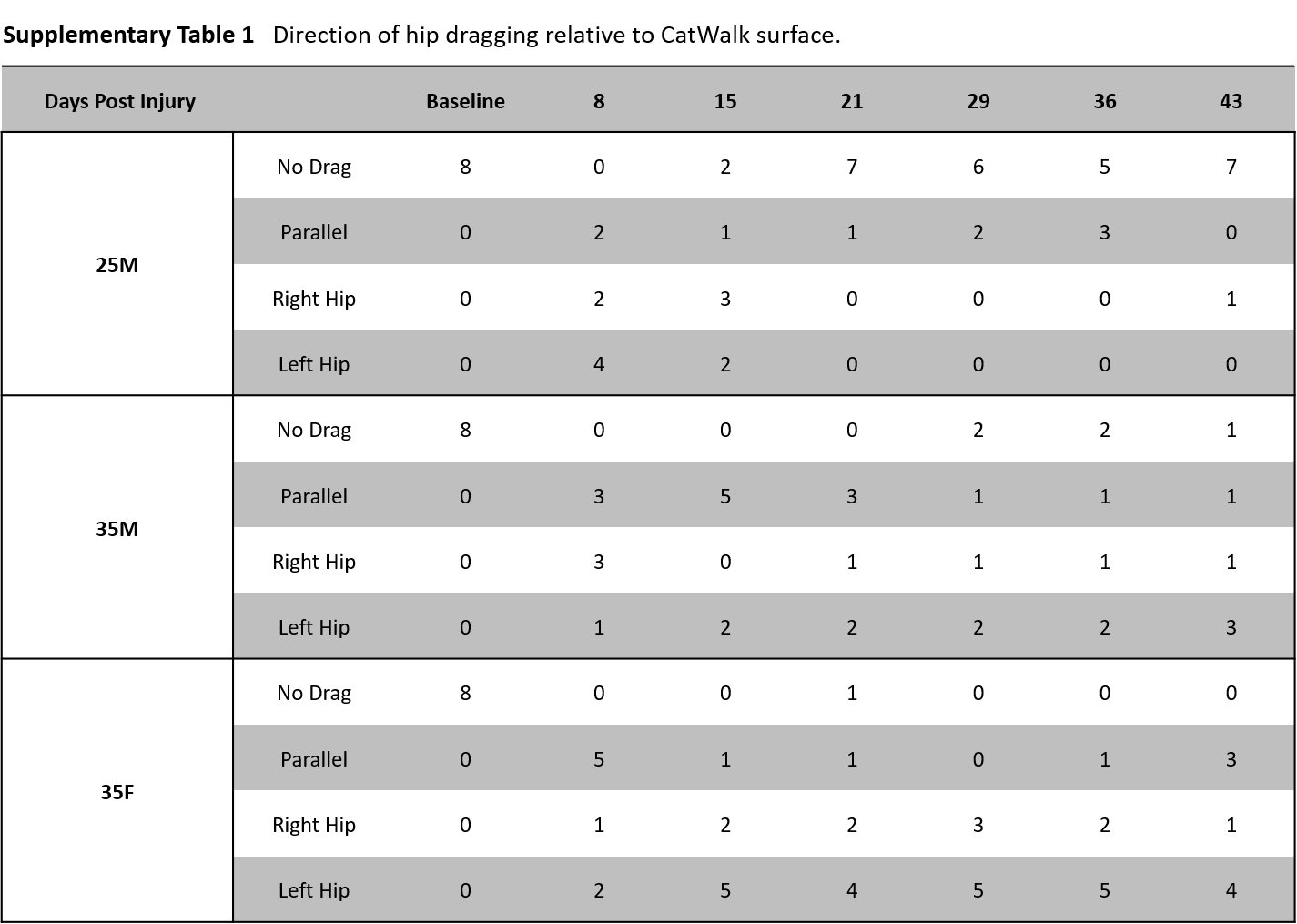

Supplement: Supplementary Table 1 — Direction of hip dragging relative to CatWalk surface. [file Table_1.DOCX]

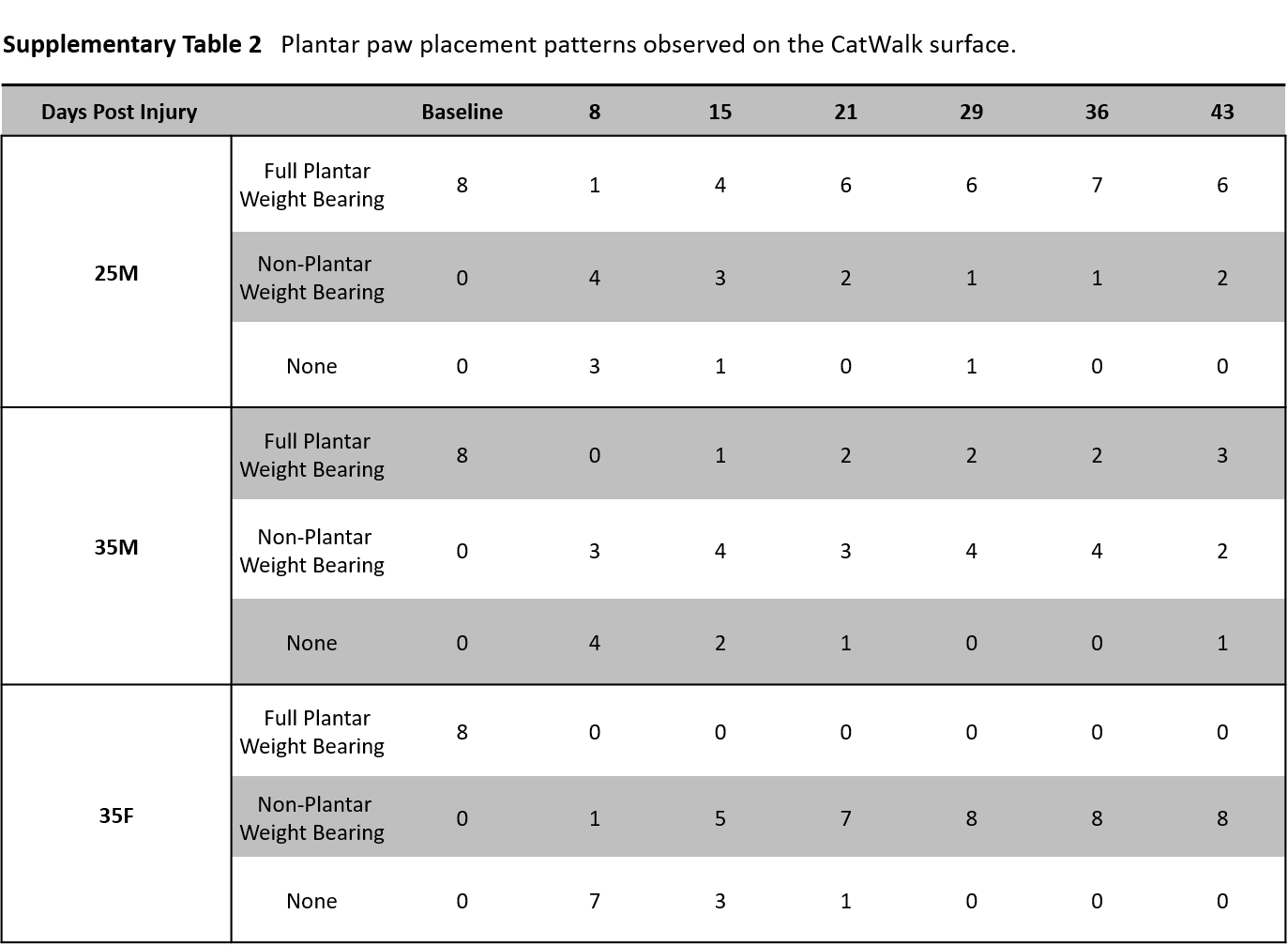

Supplement: Supplementary Table 2 — Plantar paw placement patterns observed on the CatWalk surface. [file Table_2.DOCX]

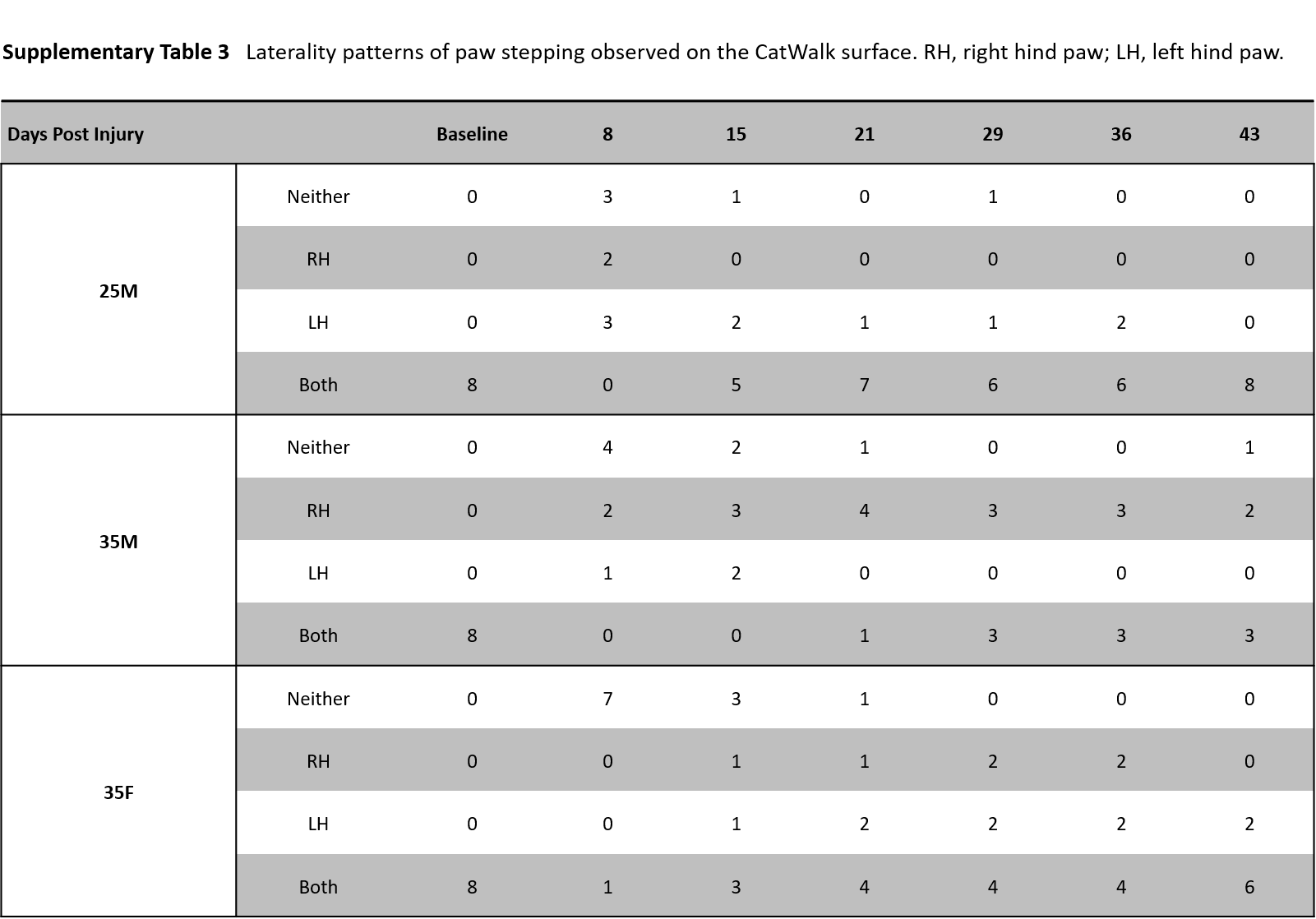

Supplement: Supplementary Table 3 — Laterality patterns of paw stepping observed on the CatWalk surface. RH, right hind paw; LH, left hind paw. [file Table_3.DOCX]
